# Antiplatelets and Vascular Dementia: A Systematic Review

**DOI:** 10.1155/2022/9780067

**Published:** 2022-09-19

**Authors:** Peter Alexander, Shakthi Visagan, Sara Jawhar, Amogh Kare, Noor Issa, Reem Issa, Abbas Jawhar, Sneha Thomas, Vasavi Gorantla

**Affiliations:** ^1^Department of Anatomical Sciences, St. George's University School of Medicine, Saint George's, Grenada; ^2^Department of Internal Medicine, University of Maryland Medical Center, Baltimore, USA

## Abstract

Vascular dementia (VD) is a neurocognitive disorder whose precise definition is still up for debate. VD generally refers to dementia that is primarily caused by cerebrovascular disease or impaired cerebral blood flow. It is a subset of vascular cognitive impairment, a class of diseases that relate any cerebrovascular injury as a causal or correlating factor for cognitive decline, most commonly seen in the elderly. Patients who present with both cognitive impairment and clinical or radiologic indications of cerebrovascular pathology should have vascular risk factors, particularly hypertension, examined and treated. While these strategies may be more effective at avoiding dementia than at ameliorating it, there is a compelling case for intensive secondary stroke prevention in these patients. Repeated stroke is related to an increased chance of cognitive decline, and poststroke dementia is connected with an increased risk of death. In general, most physicians follow recommendations for secondary stroke prevention in patients with VD, which can be accomplished by the use of antithrombotic medicines such as antiplatelets (aspirin, clopidogrel, ticlopidine, cilostazol, etc.). In individuals with a high risk of atherosclerosis and those with documented symptomatic cerebrovascular illness, antiplatelets treatment lowers the risk of stroke. While this therapy strategy of prevention and rigorous risk management has a compelling justification, there is only limited and indirect data to support it. The following systematic review examines the role of antiplatelets in the management of vascular dementia in published clinical trials and studies and comments on the current evidence available to support their use and highlights the need for further study.

## 1. Introduction

Vascular dementia (VD), a neurocognitive disorder, is the deterioration of memory and cognitive functioning resulting from a reduced or lack of blood flow to the brain. This form of dementia is the second most common cause of age-related cognitive impairment, accounting for 20% of all dementia cases [1]. Similar in symptomatology to other forms of dementia, vascular dementia begets behavioral symptoms, locomotor abnormalities, and autonomic dysfunction. Underlying cardiovascular diseases that affect blood supply, such as hypertension, high cholesterol, and heart disease, are all risk factors of this disorder [1]. Considering extended life expectancy and the increasing prevalence of uncontrolled hypertension, the worldwide incidence of patients with dementia is expected to reach 75.6 million by 2030 and rise even further to 135.5 million by 2050 [2].

VD is classified into 5 major subtypes based on its root causes. These are (i) multiple infarct dementia, a series of multiple small strokes triggering a loss of brain function, (ii) subcortical vascular dementia (small vessel disease), a single major stroke that penetrates the vessel walls of arteries leading to hippocampal damage, (iii) stroke-induced dementia, (iv) Cerebral Autosomal Dominant Arteriopathy with Subcortical Infarcts and Leukoencephalopathy (CADASIL), a rare small vessel disease caused by mutations of the Notch3 gene, and (v) mixed dementia (VD + Alzheimer's Disease (AD)), the coexistence of clinically similar symptoms of both AD and VD [1, 3]. Multiple infarct, subcortical, and stroke-induced dementia types are based on the vascular events that contribute to VD, leading to cognitive impairment, memory loss, and psychological issues. On the other hand, CADASIL refers to a genetic subtype of VD in which genetic mutations have to be present in the Notch3 gene coding for a transmembrane receptor (Notch receptor 3) on vascular smooth muscle cells. This variant is characterized by migraine with aura, stroke, and psychiatric symptoms leading to dementia and disability [4]. The ability to differentiate the various classifications of VD is crucial for dictating treatment strategies for each subtype.

At its core, the management of VD is establishing a timely diagnosis, educating, supporting, and maximizing independence and quality of life. There have been attempts at finding a successful pharmacological regimen to aid in managing VD, the most extensively studied being cholinesterase inhibitors and memantine. The philosophy behind this treatment stems from the suggested overlap between the neurological and neurochemical similarities between AD and VD. However, pure VD studies suggest that the cholinergic system within the CNS is intact [5]. These studies show a small but significant benefit for cognitive function, and they are not included in current guidelines due to the effectiveness of diagnosis and possible adverse side effects associated with common anticholinergic activity [5]. Randomized control trials studying memantine and NMDA antagonists conducted by Wilcock et al. and Orgogozo et al. showed similar results with cholinesterase inhibitors with a small but significant cognition benefit [6, 7]. There is some positive evidence for the use of cerebrolysin to improve cognition and global outcomes in vascular disease, but there are varying results, and the length of studies was short [8].

Few studies analyze primary preventative measures where cognitive benefits and global outcome improvement were primary outcomes in VD. Previously, there was little evidence for the pharmacological monotherapy of statins, blood pressure reduction, or antiplatelets in the prevention or treatment of VD. However, their use is important for the treatment of VD risk factors, and there may be some benefit in the combined preventative approach illustrated by the FINGER study. In the FINGER study, the use of vascular risk factor reduction, cognitive training, nutritional counselling, and exercise in high-risk patients has produced intriguing evidence of reduced cognitive decline [9]. Due to the nature of the disease and the lack of solid research and evidence for any one singular treatment plan for VD, prevention of this disease is critical. Risk factor manipulation is one strategy, and antiplatelet agents remain imperative for the treatment of VD risk factors such as secondary stroke and thromboembolism prevention. With regard to stroke prevention, antiplatelets such as aspirin, cilostazol, and ticlopidine and anticoagulants such as warfarin play a central role. The most common antiplatelets studied in VD treatment and primary prevention are salicylate-based therapy such as aspirin and, more recently, cilostazol and dipyridamole, which have been gaining research interest [10]. Most antiplatelet agents inhibit the body's cyclooxygenase enzymes, decreasing the level of thromboxane A2, which stimulates platelet aggregation. These agents reduce thrombi formation and the incidence of stroke, especially for those at high risk for atherosclerosis and for those with cerebrovascular disease. In patients with a history of intracranial hemorrhage or other clear contraindications to the use of antiplatelets (such as systemic bleeding), the risk of bleeding due to antiplatelets must be weighed against the risk of stroke incidence when considering antiplatelets like aspirin in the therapy for VD. Physicians who consider antiplatelets in the treatment of VD for the prevention of recurrent stroke must also consider the well-studied gastroduodenal toxicity of aspirin and other NSAIDs, the risks associated with cessation of antiplatelet therapy, and the risk of possible breakthrough stroke (the failure of antiplatelet therapy in preventing a stroke incidence). The use of antiplatelets with their potential benefits and adverse effects is included in further detail in the following review.

Antiplatelet therapy has a long-term cognitive protective benefit and appropriate care of vascular risk factors should enhance long-term cognitive prognosis in stroke patients. The following systematic review highlights the most recent empirical studies and research covering the use of antiplatelet drugs in the management of VD in the following areas: primary prevention, treatment to improve cognition, and its role in integrated therapy.

## 2. Methods

**Figure 1:**
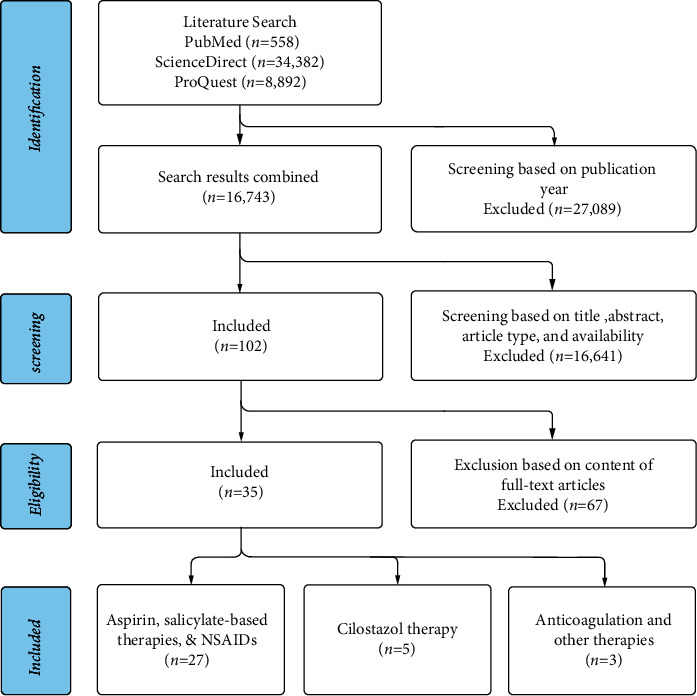
PRISMA flowchart describing the literature screening of studies concerning the use of antiplatelets in managing VD.

This systematic review strictly follows the Preferred Reporting Items for Systematic Reviews and Meta-Analyses (PRISMA) guidelines [11]. A search of the literature was done on February 10th, 2022, through the databases PubMed, ScienceDirect, and ProQuest. The queries made to elicit the search results followed the pattern of a search term referring to vascular dementia and another search term referring to a specific antiplatelet drug. The three possible search terms for the vascular dementia pathology were “vascular dementia,” “vascular cognitive deficit,” or “vascular cognitive impairment.” Seven search terms were used for the set of antiplatelet drugs: “antiplatelet,” “antithrombotic,” “aspirin,” “clopidogrel,” “plavix,” “ticlopidine,” or “cilostazol.” Permuting the query by combining one of the vascular dementia keywords with one of the antiplatelet drug keywords yields 21 unique searches that were each done on the three databases. This initial search yielded 43,832 articles. Articles published before 2000 were then excluded leaving 16,743 articles. Regardless of our best efforts and a comprehensive search, we acknowledge that not all relevant research works and studies may have been included in this review and some articles may be inadvertently omitted. Inclusion and exclusion criteria were applied and only relevant research works regarding our research question were considered. A total of 35 articles were kept (Figure 1) [12–46].

### 2.1. Inclusion Criteria

Study selection included the following criteria: studies written in English and conducted on humans, in the last 22 years (articles published between 2000 and 2022 were included), that were relevant to our topic and research question (the use of antiplatelets in the treatment or management of vascular dementia or vascular-related cognitive impairment or vascular cognitive deficit), peer-reviewed, and full texts, including these study types: clinical trials and observation studies (case-control, cohort, and cross-sectional studies).

### 2.2. Exclusion Criteria

Duplicates of articles, papers not published in English, books and book chapters, letters to the editor, opinionated articles, editorials, or letters, and in vitro or animal experiments were eliminated from the literature review. Articles that were purely abstracts or poster presentations were excluded. Narrative and systematic reviews, meta-analyses, and other literature reviews were omitted. Articles detailing the use of antiplatelets primarily for the treatment of a condition other than VD were eliminated. This method is summarized in Figure 1.

Screening of the literature has been done mirroring the protocol described in the PRISMA statement [11]. A total of 35 articles were found. 27 articles discussed aspirin, salicylates, or NSAIDs as the main intervention aimed at reducing cognitive decline in those with vascular dementia. 5 articles investigated cilostazol for the same purpose or compared cilostazol against aspirin regarding their neuroprotective effects. Only 3 articles were found discussing the use of anticoagulants and other related therapies for the treatment of vascular cognitive deficit.

## 3. Results

In total, 43,832 publications were found, 558 from PubMed, 34,382 from ScienceDirect, and 8,892 from ProQuest. After excluding articles published before 2000, articles were then manually screened based on title, abstract content, article type, and availability, leaving 102 articles to be checked for eligibility. Given that antiplatelet therapy and its use in treating vascular dementia pale in comparison to its use in treating disorders of coagulation and that vascular dementia is not as commonly discussed relative to other primary dementias (such as AD), the number of articles and studies found is low. Many articles note this disparity and encourage the running of trials and studies to elucidate this topic further. We also note that although ticlopidine is an important consideration as an antiplatelet for the treatment of certain vascular pathologies, we did not find any recent trials or studies examining its use for the management of VD within the past 22 years. This is likely due to newer drugs like plavix or clopidogrel gaining popularity over ticlopidine for managing similar clinical scenarios

Table 1 shows studies on the use of differing antiplatelet and related therapies for various vascular-related cognitive decline pathologies within the past 22 years.

## 4. Discussion

### 4.1. Vascular and Alzheimer's Dementia

A discussion of the management of VD would not be complete without noting that, due to VD's poor diagnostic criteria, it can often be misdiagnosed as AD. Clinically, AD shares much of its symptomology, and there is a clinical need not only to depend on the presence of a cognitive disorder but also to rule in a vascular cause and rule out any alternative pathologies [47]. Screening tests usually used for dementias such as the mini-mental state examination are not as useful for vascular dementias. This is because the cognitive impairment that occurs in VD varies more than other dementias depending on where the specific vascular pathology is located. Instead, screening tests like the Montreal cognitive assessment scale or VADAS-cog are more applicable for populations with vascular dementia due to their sensitivity to attention and executive function [5]. Imaging the brain (MRI and CT) is standard for diagnosis of dementia; however, a substantial level of cerebrovascular disease is needed to establish vascular dementia as the cause [5]. For this reason, the diagnostic criteria are being modified to include the use of genetic markers and biomarkers [48]. Most genetic studies are being attributed to chance findings; however, there seems to be an association between high homocysteine concentrations and vascular dementias, which can be explained by MTHFR polymorphisms [5]. Unfortunately, some studies have also shown that there can be an interplay between the use of antiplatelets in the treatment of dementia and the risk of developing AD. Chang et al. (2016) showed that although there was no benefit in nondementia Alzheimer's patients who took aspirin 40 mg daily, patients with type 2 diabetes who took aspirin at a dose of 80 mg or more per day had an increased risk of developing Alzheimer's dementia. The mechanism of these effects, however, needs to be clarified [34]. Some suggest that amyloid angiopathy, seen in AD, can lead to cerebral microbleeds with the administration of salicylates and is associated with the development of dementia [35]. Others have noted that the common genetic biomarker, the APOE epsilon 4 allele, is an important association between AD and VD. Szekely et al. (2008) found that NSAID users had a reduced risk of AD, but this was only present in those positive for the APOE epsilon 4 allele [26].

### 4.2. Common Antiplatelets Used in the Treatment of VD

In general, antithrombotic therapy given for patients with VD is usually reserved for those with a history or at high of stroke or transient ischemic attack. While there are a variety of antiplatelet regimens used to treat vascular dementia, two commonly used drugs are aspirin and cilostazol. Both reduce the occurrence of blood clots; however, they have different mechanisms of action. Aspirin (acetylsalicylic acid) chiefly exerts its effects by suppressing the production of prostaglandins and thromboxane. It does so by irreversible inhibition of the cyclooxygenase (COX) enzymes 1 and 2. While accomplishing an anti-inflammatory effect by reducing the production of prostaglandins, aspirin also decreases the aggregation of platelets via suppressing the output of thromboxane [39]. Though the reasoning for administering such a therapy is strong and can be obvious given the pharmacology of antiplatelet agents and the pathology behind vascular risk factors for dementia, the evidence supporting it is limited. For example, the ASPREE trial showed that low-dose aspirin had no effect on dementia incidence. On average, the treatment groups did not benefit from aspirin when it came to clinically probable or possible Alzheimer's Disease, mild cognitive impairment, or cognitive decline. Treatment effects did not differ between subgroups defined by age, sex, ethnicity, health, or prior NSAID use. Because of the rigor of this large double-blinded placebo-controlled trial, this study provides strong evidence that starting low-dose aspirin and continuing for almost 5 years does not reduce the risk of cognitive decline, dementia, or AD in older adults [42]. Furthermore, some studies, such as that by Wichmann et al. (2016), examined NSAID use and incident cognitive impairment over a 20-year period and found no evidence of a protective effect. However, it is difficult to remove all biases, highlighted by Devine et al., where they show that patients that are prescribed aspirin had significantly higher rates of vascular disease. They state that, in those with dementia living with care-taker, aspirin has a small protective effect prolonging institutionalization to death time [14]. Aspirin users may be at increased risk of cognitive impairment, possibly reflecting underlying relationships between aspirin indications and cognitive impairment risk. Waldstein et al. (2010) examined in greater detail what aspects of cognition were protected with the use of NSAIDs and found that aspirin was found to be related to less decline on tests that assessed memory, concentration, and visual memory. NSAIDs not only showed a similar profile of improvement but also showed a greater increase in decline on other measures of cognitive function. Though they note that these effect sizes were small, their results add to the debate on the true effectiveness of aspirin and NSAIDs in the use of vascular cognitive decline [27]. Although chronic inflammation has been linked to dementia, some inflammatory responses are beneficial, so suppressing them with anti-inflammatory drugs may be less effective than promoting or preserving a dynamic, balanced response [33]. Other studies, however, do show some indirect or at least limited evidence for the use of aspirin for the treatment of dementia in certain subgroups. For example, low-dose aspirin may reduce dementia risk in type 2 diabetes mellitus (T2DM) women but not in men; larger sample sizes and longer follow-up periods with genetic and sociocultural evaluation of participants are needed to confirm these findings, which may be biased due to the cardiovascular benefit offered by aspirin and the benefits it offers to T2DM patients [41]. Similar findings were shown in a population of nondemented Swedish elderly women with cardiovascular risk by Kern et al. (2012) in that women on low-dose aspirin performed better on MMSE than those not on aspirin [28]. Zhang et al. (2013) also show that aspirin is neuroprotective in those with acute ischemic stroke up to 3 months after stroke [31]. An important consideration to aspirin use in VD is the tradeoffs between the minor benefits in cognitive decline and the elevated bleeding risk and the compliance issues in elderly populations, especially those with dementia [20]. One avenue of NSAID use for the management of vascular cognitive decline was the use of COX-2 selective inhibitors such as celecoxib and rofecoxib but results were contradictory among several studies [18, 21, 25]. Debate rages over NSAID use in general for managing dementia because most observational studies yield contradictory results.

Another frequently used drug, cilostazol, is a reversible phosphodiesterase III inhibitor resulting in vasodilation and platelet aggregation inhibition. Cilostazol reduces the rate of cyclic adenosine monophosphate (cAMP) degradation, thereby increasing the level of cAMP in cells. Within platelets, a higher cAMP level increases protein kinase A activity, inhibiting activation and aggregation [44]. The decrease in platelet aggregation reduces the chance of clot formation, thus decreasing the chance of a stroke caused by aberrant clotting. Huang et al. (2021) developed a pilot study to examine cilostazol as a possible treatment for poststroke cognitive decline. They found that cilostazol may be considered “noninferior” for poststroke cognitive change despite no significant differences in overall outcome between cilostazol and control groups after 6 months. The cilostazol group had a higher rate of peripheral arterial occlusive disease (PAOD) and might account for the inherent bias in the improvement in cognitive change; that is, cilostazol is recommended by Taiwanese national health insurance guidelines for treating PAOD symptoms of intermittent claudication and thus the cilostazol group received more PAOD patients. Their results suggest that a randomized case control study should be conducted in the future regarding cilostazol as a treatment for cognitive decline after stroke; ultimately, for cilostazol to be considered effective in treating postischemic cognitive impairment, large-scale double-blind studies are required [44]. In other retrospective studies, cilostazol has been shown to improve MMSE scores relative to those that were not taking cilostazol, but this effect was nonsignificant [29]. The neuroprotective effect of cilostazol and the motivation for many of these studies come from the idea that cilostazol, like aspirin, can reduce the accumulation of amyloid-beta and improve cerebral circulation [29]. Recently, cilostazol has also been introduced as part of the management for cerebral small vessel diseases. Kim et al. (2021) investigated the use of cilostazol for reducing white matter changes due to lacunar infarction relative to aspirin. They also examined its effect on the number of lacunes and cerebral microbleeds. Although they found that there was no significant difference between cilostazol and aspirin on white matter hyperintensities, they did note that cilostazol does significantly reduce the chance of ischemic cerebrovascular injury relative to aspirin [46]. White matter hyperintensities are a relatively new biomarker being investigated in cerebral small vessel diseases and, in general, cerebrovascular pathologies. As brain imaging with MRI becomes more feasible and quicker to perform, WMH burden might be investigated as a possible marker of cognitive decline and increased cardiovascular risk. The use of WMH in assessing cerebrovascular risk will also be greatly improved with the introduction of better software and algorithms for image analysis and segmentation of the lesions found in imaging. For example, in the work of Khezrian et al. (2020), they found that aspirin moderates the negative effects of WMH burden and that there is a link (no establishment of causality, however) between WMH lesion volume, cardiovascular risk, and cognitive decline. They also highlight earlier hypotheses found in aspirin studies that, in older populations taking aspirin, cognitive decline may follow a different, relatively unknown etiopathogenesis, one that can bypass the neuroprotective anti-inflammatory effects of aspirin and similar medications [43].

Antithrombotic therapy with antiplatelets is not without risks. One well-studied and obvious risk associated with the use of aspirin and other NSAIDs in the treatment of VD and recurrent stroke is gastroduodenal toxicity. Aspirin and NSAIDs prevent the synthesis of prostaglandins in the gastric lumen, which have a protective effect by increasing the secretion of mucus and bicarbonate secretion and also decreasing acid secretion. If physicians are worried about gastroduodenal toxicity, namely, upper gastrointestinal tract bleeding and ulcers, and would like to prevent an incidence of gastritis, then proton-pump inhibitors like omeprazole or prostaglandin analogues such as misoprostol can be concomitantly administered [49]. Another clear risk associated with the use of aspirins is the increased risk of bleeding and, in particular, the increased risk of cerebral hemorrhage and cerebral hypoperfusion. In the ASPREE trial, it was found that daily low-dose aspirin did not improve disability-free survival but increased the risk of major hemorrhage [42]. This risk can also be associated with the presence of breakthrough stroke or the failure of antiplatelet therapy in preventing recurrent stroke. For example, it has been shown that the combination treatment of clopidogrel and aspirin was unlikely to prevent stroke and paradoxically increased bleeding and mortality [50]. The preliminary results of the Secondary Prevention of Small Subcortical Strokes (SPS3) [51] do not support the use of clopidogrel and aspirin for the management of recurrent stroke in those with subcortical strokes. Other combination therapies, such as aspirin with dipyridamole or monotherapy with either ticlopidine or clopidogrel, have proven to be more effective at preventing recurrent stroke relative to aspirin alone [52–54].

### 4.3. The Use of Antiplatelets and Anticoagulants in the Primary Prevention of VD

The management of VD is complex, and the role of antiplatelet therapy is uncertain but can play an important role in its prevention. Oveisghara et al. (2016) found no link between salicylate use and dementia incidence among normal cognition participants but found a link among a subgroup of normal cognition participants who scored poorly on neuropsychological tests. These tests, with scores that were significantly different only in VD patients and not in AD patients, may be indicative of vascular brain diseases. Salicylate use may be linked with a lower incidence of dementia among participants who are more prone to develop dementia [35]. This finding provides a strong base for the use of antiplatelets in the prevention of VD in its early stages. The significance of NSAID use (including aspirin) to improve cognitive function varies between studies depending on a number of variables including primary and secondary outcome measures, access to pharmacology, and prevalence of comorbidities. However, in general, studies have found a net benefit in cognition due to the anti-inflammatory effects and a risk reduction in developing VD attributed to the antiplatelet properties [12, 13, 15–17]. One large cohort study conducted on women found no overall improvement in cognitive function between aspirin and placebo group; however, in a secondary outcome measure, the aspirin group performed better in the category fluency test which tests an area of cognitive function highly dependent on the presence of vascular disease [19]. A more focused study on rofecoxib showed this selective COX-2 inhibitor had no impact and may even accelerate MCI to AD attributing this to the drug's tendency to raise blood pressure [18]. Recent research indicates that antiplatelets play a preventive role in lowering the risk of stroke and vascular complications. A population-based cohort study done by Ding et al. showed that 6-year follow-up patients with atrial fibrillation treated with antiplatelets were not associated with an increase in dementia risk (HR = 1.85). However, in the same study, anticoagulant therapy was associated with a 60% risk reduction of dementia (HR = 0.40) [38]. In a large claims database study conducted by Tai et al., in 2017, the use of cilostazol and the risk reduction of incident dementia were assessed, finding a decrease in incident dementia in a dose-dependent pattern (aHR = 0.75) [36]. Recently, there is positive evidence for aspirin therapy in patients with late-onset dementia (LOD). A study done by Ya-Hsu et al. found a lower incidence of subsequent incident dementia in patients with LOD who used aspirin compared to that in nonaspirin users (HR = 0.73) [39]. On the contrary, the ADAPT study found no evidence that naproxen or celecoxib has protective effects on cognitive testing, additionally, to see any benefits NSAID use must begin years before the development of dementia symptoms [21]. Moreover, a study using neuropathology autopsies found no correlation between NSAID use and an improvement in cognitive function compared to nonaspirin use [22]. Another hit to the use of aspirin comes from a randomized double-blind placebo-controlled study called the PRoFESS trial where they found the use of neither aspirin, extended-release dipyridamole, or clopidogrel and telmisartan are neuroprotective after a stroke [23]. Some studies have investigated the use of dual antiplatelet therapy, that is, the coadministration of both aspirin and clopidogrel. In the work of Pearce et al. (2014), they found that cognitive function was not dependent on lowering of blood pressure or by the dual therapy, but they do note that their population, though mainly diagnosed with lacunar infarcts, did not have high rates of cognitive decline to begin with.

Despite the contradictory evidence, it is important, when considering aspirin, to maintain awareness of aspirin resistance (AR) that may develop in patients. A study by Staszewski et al. showed AR is not uncommon in patients with cerebral small vessel disease and VD, at 24-month follow-up, patients with AR ischemic strokes occur at higher frequency (OR = 3.1) as well as the radiologic progression of disease (OR = 2.2) highlighting the necessity for AR screening and encouraging the importance of compliance [37]. Antiplatelets like aspirin and cilostazol have a body of favorable evidence for primary prevention of VD; however, anticoagulants may be more effective. Recently, direct oral anticoagulants (DOACs) have been used to lower the risk of dementia in patients with atrial fibrillation. Lee et al. (2021) examined the use of four DOACs, rivaroxaban, dabigatran, apixaban, and edoxaban, and compared the risk of dementia with respect to warfarin. They found that DOACs showed a similar risk of dementia but DOACs were more beneficial than warfarin in stroke patients who were aged 65 to 74 years [45]. Anticoagulation is effective in slowing cognitive decline because it reduces the risk of cerebral thrombosis and microembolism. The reduction in dementia risk in anticoagulant-treated AF patients could reduce cognitive impairment by up to 20% [40].

### 4.4. Benefits of Antiplatelet Therapy on Cognition in Patients with VD

The cognitive impairment following a stroke has a significant burden on society. For this reason, finding solutions or factors that can decrease this cognitive impairment is of great importance. The decrease in cognitive function resulting from vascular dementia is usually due to a lack of perfusion to certain brain areas. Antiplatelets inhibit thrombus formation and reduce the blood flow to different regions of the brain, potentially reducing the severity of cognitive impairment. Certain drugs like cilostazol have vasodilatory effects and protective effects on the endothelium in addition to their antiplatelet effect; therefore, some VD patients may benefit from their use [55]. A recent clinical trial supported this, reporting a decreased risk of repeated stroke when treated with cilostazol, especially hemorrhagic stroke [56]. Additionally, a study conducted on rats showed that cilostazol prevented cerebral hypoperfusion-induced white matter damage and cognitive impairment, which shows its promise as a treatment for cognitive impairment experienced by vascular dementia [36]. There are limited studies about the direct benefit between VD's cognitive decline and antiplatelets; however, it was seen that people with atrial fibrillation treated with antiplatelets were less likely to develop vascular dementia [38].

### 4.5. The Role of Antiplatelets in Combination Therapy for VD

Antiplatelet therapy has proven to be useful in different dual drug therapies in short-term use and lifestyle integrated combination therapies to reduce the risk of stroke and, therefore, the risk of vascular dementia. A 2018 study was conducted by first analyzing the data from Norfolk and Norwich University Hospital Stroke Register of 3,572 patients admitted for an ischemic stroke between 2003 and 2015 and then placing them on three different antiplatelet regimens. The first group was treated with aspirin monotherapy, the second with clopidogrel, and the third was placed on aspirin and dipyridamole combination therapy. The study revealed a much better short-term outcome with a 38% relative risk (RR) reduction in mortality within 3 months of discharge in patients placed on the combination therapy of aspirin and dipyridamole as compared to those placed on monotherapies. In addition, there was no significant increase in adverse consequences linked to the short-term use of dual therapy. However, solitary clopidogrel use proved more beneficial in the long term with a 61% RR reduction in mortality from 1–3 years compared to solitary aspirin use and combination therapy of aspirin and dipyridamole. These results supported placing patients on short-term dual drug therapy of aspirin and dipyridamole for 12 months and then switching to a long-term clopidogrel monotherapy for the best outcomes and least risk of mortality as similarly recommended for patients with acute coronary syndromes [57]. For the treatment of dementia, cilostazol is also sometimes coadministered with donepezil and has been shown to be neuroprotective in mild cases but not in those with moderate to severe dementia [30].

In addition, the treatment of vascular dementia has recently taken a different approach than ordinary standardized care, such as the example of the FINGER study. Multiple therapies and lifestyle changes are being integrated into preventative measures of vascular dementia such as cognitive training, positive awareness and changes in diet, therapy for vascular risk reduction, and even exercise. This multidimensional approach shows great promise in high-risk individuals to reduce the onset of vascular dementia. Epidemiological cohort studies have shown a decline in overall dementia, and although the reason for that is not fully known, it is promising to assume that this decrease is due to the reduction of vascular risk [2].

## 5. Conclusion

Vascular dementia is a progressive cognitive disease that impacts the quality of life of mainly elderly individuals. The progression of VD is tightly correlated to cerebrovascular events and the cognitive impairment that follows. In contrast to AD, where there are links to amyloidosis and other possible pathogenic factors, prevention of the disease is not well known. VD presents similarly to AD, and differentiating the two is challenging; however, screening tests like the Montreal cognitive assessment scale or VADAS-cog are specific to vascular dementia due to their high sensitivity compared to the mini-mental state examination, the standard for diagnosing AD. Diagnostic criteria are advancing to more sophisticated methods like molecular genetic markers, which allow for an increasingly specific diagnosis of vascular dementia. Antiplatelet therapy, specifically aspirin and cilostazol, and anticoagulant therapy with warfarin have been suspected of preventing and slowing down the progression of vascular dementia after a correct diagnosis has been made. Both aspirin and cilostazol inhibit platelet aggregation in various ways: aspirin inhibiting the cyclooxygenase (COX) enzyme and cilostazol inhibiting phosphodiesterase III inhibitor, leading to a decrease in stroke and vascular diseases. Antiplatelet therapy such as that with cilostazol is also believed to alleviate the risk of cognitive deterioration that is associated with vascular dementia. This is primarily because they inhibit thrombus formation and have vasodilatory action, thus preventing the occlusion of vessels and reducing ischemic events in the brain. Patients placed on antiplatelet combination short-term therapy of aspirin and dipyridamole have also shown a reduced relative risk in mortality without increasing adverse effects. Patients with a high risk of developing VD may benefit more from the addition of long-term clopidogrel monotherapy. Studies involving antiplatelet therapy in the context of primary preventing or possibly treating vascular dementia are scarce. Further investigation with extended randomized control trials is required to better understand the correlation between the two. However, with our current understanding, it is safe to assume that antiplatelet therapy, at the very least, can play a beneficial role as a preventative measure in patients with a risk of VD.

## 6. Limitations

Some important limitations must be noted. Namely, this is a systematic review of recent literature and this review could have been greatly strengthened with statistical and meta-analyses of the presented studies and a deeper examination of the types of treatment involved; that is, for each treatment option presented, such as aspirin, cilostazol, dipyridamole, and DOACs, an assessment of the amount of variation between the results of the studies could have been examined. An obstacle to such an analysis, however, would be the fact that few large, double-blind, random controlled trials for the treatment of vascular dementia and vascular cognitive impairment with antiplatelet agents exist. One reason for the lack of studies would be the fact that antiplatelet treatment is usually considered for those with VD and a concomitant history of stroke or transient ischemic attack. Though stroke is a major risk factor for VD, the main vascular risk factor for VD is hypertension. Furthermore, this treatment, as emphasized earlier, mostly shows efficacy in preventing secondary stroke and only indirectly improves cognitive outcomes. With the many adverse side effects that antiplatelet agents may cause, such as increased bleeding, upper GI toxicity, and intracerebral hemorrhage, physicians often do not administer these agents to the common set of patients who suffer from VD, usually those who are older than 65 years and are contraindicated for antiplatelets due to these very same adverse effects. Another limitation of this review, and studies involving antithrombotic therapy for VD in general, is that the standards for cognitive improvement are sometimes ill-defined or are too broad and can be generous, thus favoring the use of antiplatelets. For example, antiplatelets are well known to have great cardioembolic protective benefits and are used in many diseases ranging from T2DM and PAOD. Improving outcomes in those diseases tends to improve cognitive outcomes as well, even though the benefit is indirect, thus confounding the results of cognitive improvement seen with antiplatelets. This review notes this bias exists but further statistical methods are required to correct for it. These shortcomings can also be overcome with a better stratification of cognitive outcomes that focus directly on the relationship between vascular risk factors and cognitive outcomes. Some other limitations of this review are that only studies in the past 22 years were included and that only PubMed, ScienceDirect, and ProQuest were searched. More studies from more diverse origins could have been included in a broader search. Finally, this study did not address dose, frequency, or specific medications or formulations of the treatments examined.

## Figures and Tables

**Table 1:** Studies on the use of antiplatelets for management of vascular dementia.

	Authors	Country	Intervention	Study design and population	Findings and conclusions
1	Jonker et al. [12]	Amsterdam, Netherlands	NSAIDs (including aspirin) (*n* = 137) versus control (no NSAIDs) (*n* = 475)	Population-based study (*n* = 612) via the Longitudinal Aging Study Amsterdam (LASA)	For a population with an age range varying from 62 to 85, this study found no differences between users and nonusers of NSAIDs in mean MMSE scores. Also no differences were found between individual subscores on items such as recall (both immediate and delayed) and coding tasks between users and nonusers. However, with regard to a decline in memory, NSAIDs seem to lower the risk. This protective effect was more associated with the use of aspirin and no other NSAIDs. They suggest that the additional antiplatelet properties of aspirin might contribute to its protective effects.

2	Kang and Grodstein [13]	USA	NSAIDs (including aspirin) (*n* = 11,216) versus control (no NSAIDs) (*n* = 8,749)	Prospective cohort study (*n* = 19,965) via the Nurses' Health Study cohort of women aged 70–81	For women between the ages of 70 and 81, this study found that NSAID use has a substantial effect on cognitive function and decline. Long-term users of nonaspirin NSAIDs showed marginally better overall performance on tests measuring cognitive decline (verbal fluency, delayed recall, East Boston Memory Test, TICS, etc.) relative to those who do not use NSAIDs. The authors postulate that the protective effects of nonaspirin NSAIDs against cognitive decline might be due to the inhibition of cyclooxygenase mediated inflammation and the decrease of amyloid-beta protein, an important factor in Alzheimer's Disease pathogenesis. They also state that the use of NSAIDs reduces the risk of cerebrovascular pathologies and vascular dementia.

3	Devine and Rands. [14]	London, UK	Aspirin (*n* = 38) 75 mg/day (*n* = 28) and 150 mg/day (*n* = 10) versus control (no aspirin) (*n* = 40)	Retrospective case-notes analysis (*n* = 78) of patients with ischemic VD	This study primarily highlights the difficulty in removing indication bias in studies involving the efficacy of aspirin in those with VD; that is, aspirin in those with VD might be indicated and prescribed for patients with other copresenting conditions; most of the time, including in this study, those in the aspirin group had significantly higher rates of vascular disease compared to the nonaspirin group (not necessarily due to the use of aspirin itself). In this study with a small sample size, it was shown that aspirin has a small protective effect (improves survival times to institutionalization and to death) in those with dementia and with those who lived with a care-taker. They argue that this is due to better compliance relative to those who live alone with dementia, where compliance with aspirin may not be guaranteed. The authors also note that this effect is not that robust when accounting for the lower mean age of the patients prescribed aspirin and that larger studies are needed.

4	Nilsson et al. [15]	Sweden	Aspirin (*n* = 175) versus paracetamol (*n* = 143) versus NSAID (not including aspirin or paracetamol) (*n* = 54) versus D-propoxyphene (*n* = 69) versus control (*n* = 261)	Population-based study (*n* = 702 or 351 twin pairs) via the Swedish longitudinal study “Origins of Variance in the Old-Old : Octogenarian Twins” (OCTO-Twin)	This study found that aspirin might reduce the risk of Alzheimer's Disease (AD). Both nonaspirin NSAIDs and aspirin showed a numerical reduced risk of AD and cognitive decline, even after correcting for stroke, TIA, myocardial infarction, angina pectoris, and congestive heart failure, but this reduction was statistically insignificant. The authors also note that, in all studies concerning the efficacy of aspirin in reducing dementia risk, the use of over-the-counter aspirin and the use of prescribed aspirin must both be analyzed. They also suggest that access to pharmacotherapy in patients with dementia differs from that in those without dementia which must also be accounted for. They further support the idea that the protective effects of NSAIDs and aspirin come from COX-1 inhibition and slowing down of the inflammatory processes that contribute to AD development.

5	Cornelius et al. [16]	Stockholm, Sweden	Aspirin (*n* = 154) versus NSAIDs (not including aspirin) (*n* = 76) versus control (*n* = 1,079)	Population-based study (*n* = 1,299) via the longitudinal Kungsholmen Project	The apoE epsilon4 allele has been found to be a risk factor for both AD and other cerebrovascular diseases and this study aimed to find an association between dementia, the use of NSAIDs, and the apoE apsilon4 allele. This study found that those who were on aspirin and did not have the apoE epsilon4 allele had an increased risk of AD. They also note that this finding persists even after correcting for other possible underlying diseases and indications. This study additionally found that there is a small protective effect of NSAIDs against AD in general, but this was not statistically significant. The authors postulate that dementia in those who are negative for the apoE epsilon4 allele may not owe its pathogenesis to inflammatory reactions but through some other unknown mechanism. Similarly, the protective effects of NSAIDs in the elderly population might be due to protection against other comorbidities and vascular changes and not necessarily due to the anti-inflammatory effects and the prevention of AD.

6	Shepherd et al. [17]	Sydney, Australia	Aspirin (*n* = 69) versus control (no aspirin) (*n* = 82)	Population-based study (*n* = 151) via the Sydney Older Persons Study (SOPS)	This study aimed to find an association between human leukocyte antigen (HLA-DR) genotype, aspirin use, and performance in cognitive testing. This study supports the protective effect aspirin has on cognitive performance. This effect was also seen in individuals with the HLA-DRB1∗01 allele. The presence of the DRB1∗05 allele was associated with reduced cognitive performance. The study also suggests that there is no interaction between aspirin use, HLA-DR genotype, and cognitive performance. The authors postulate that the protective effects of aspirin are twofold: anti-inflammatory effects (which they consider might not be the primary mode of protection due to similar protective effects seen with aspirin at both low and high doses) and the prevention of cerebral vascular damage or increased cerebral perfusion due to aspirin's antiplatelet effects.

7	Thal et al. [18]	USA	Rofecoxib 25 mg (*n* = 725) versus placebo (*n* = 732)	Randomized, double-blind, placebo-controlled study (*n* = 1,457) comparing rofecoxib and placebo	This study found that rofecoxib, a selective COX-2 inhibitor, does not delay a diagnosis or the progression of AD. This finding also suggests that the inhibition of COX-2 is not a useful therapeutic marker for AD and that COX-2 is not an important factor in AD pathogenesis. One nonintuitive finding from this study is that rofecoxib might lead to increased conversion from MCI to AD, but the authors suggest this is more likely a chance finding or is due to the selective NSAID's ability to increase blood pressure (but no evidence was found for this hypothesis).

8	Kang et al. [19]	USA	Aspirin (*n* = 3,215) versus placebo (*n* = 3,162)	Observational cohort study (*n* = 6,377) via the Women's Health Study (a randomized, double-blind, placebo-controlled study investigating the use of aspirin for the primary prevention of cardiovascular disease)	For women older than 65 years receiving treatment for nearly 10 years, this study found similar cognitive performance between those taking aspirin and those receiving placebo. This null finding was shown at all three timepoints of assessment into the study, as well as in the 3 to 6 years following the study when measuring average cognitive decline. The authors note that, in the final assessment, the aspirin group performed better in the category fluency test, a partial assessment of executive function which is a cognitive system that is heavily dependent on the presence of vascular disease, but disclaim that this was the only assessment that tested executive function changes and was not a primary outcome. This study also found that subjects who had high cholesterol or who were smokers experienced less cognitive decline than those who were receiving placebo. This protective effect was not found in those without hyperlipidemia or those who were not smokers.

9	AD2000 Collaborative Group et al. [20]	UK	Aspirin (*n* = 156) versus control (no aspirin) (*n* = 154)	Randomized, double-blind, placebo-controlled study (*n* = 310) comparing aspirin and placebo (the AD2000 trial)	This study originally aimed to find the efficacy of donepezil in the management of AD. Additionally, the authors wanted to test the protective effects of aspirin in patients with AD. This study found that, for patients with AD and with no other cardiovascular disease, low-dose aspirin does improve cognitive functioning but only marginally (half-point improvement on the MMSE). The study's authors argue that the follow-up time was short (2 years) and if a longer time was considered, then the effect of aspirin may have been more significant. However, they note the tradeoff between longer follow-up and reduced compliance and drop-off, especially when considering subjects of elderly age with dementia. Considering the tradeoffs, in addition to the increased hemorrhagic risk of long-term aspirin use, the authors conclude that although some evidence exists that aspirin might slow cognitive decline, the risks outweigh the benefits and aspirin cannot always be recommended.

10	ADAPT Research Group et al. [21]	USA	Celecoxib (*n* = 726) versus naproxen (*n* = 719) versus placebo (*n* = 1,083)	Randomized, placebo-controlled study (*n* = 2,528) comparing celecoxib, naproxen, and placebo (primary prevention trial, the Alzheimer's Disease Anti-Inflammatory Prevention Trial (ADAPT))	The primary prevention trial ADAPT sought to evaluate the use of NSAIDs, such as naproxen (a nonselective COX-1 and COX-2 inhibitor) and celecoxib (a selective COX-2 inhibitor), for the primary prevention of AD. The motivation for this study comes from in vitro and in vivo mouse models that show that some NSAIDs (not necessarily celecoxib or naproxen) lower the level of amyloid-beta protein and the epidemiological evidence of lower incidence of dementia in people taking NSAIDs. ADAPT found that NSAIDs do not show a protective effect and may also lower the performance on cognitive testing. The scores on cognitive assessments were significantly lower for those taking naproxen (but not with celecoxib) when compared with placebo. Some treatments in ADAPT were terminated early due to the increased cardiovascular risk associated with celecoxib. They suggest that the protective anti-inflammatory effects of NSAIDs are only advantageous when given several years before the development of dementia symptoms and not when given close to diagnosis of MCI or AD. The authors conclude that naproxen and celecoxib should not be used for the prevention of AD.

11	Arvanitakis et al. [22]	USA	Aspirin (*n* = 389) versus nonaspirin NSAID (*n* = 215) versus control (no NSAIDs or aspirin) (*n* = 1,434)	Population-based study (*n* = 1,019) via the Religious Orders Study	This study found that NSAID use, either ibuprofen or aspirin, had no relationship with incident AD or changes in cognitive functioning. In the 325 deceased subjects that had associated neuropathology autopsies, the authors again found that NSAID use did not correlate with any measures of AD pathology. The data presented in this study can be considered to be stronger than previous investigations examining NSAID use and cognitive decline because of the extensive follow-up conducted, but the authors maintain that NSAIDs are not related to reduced cognitive decline.

12	Diener et al. [23]	35 countries worldwide	Aspirin 25 mg and extended-release dipyridamole 200 mg (*n* = 9,382) versus clopidogrel 75 mg (*n* = 9,430) (each given with either telmisartan 80 mg or placebo)	Randomized, double-blind, placebo-controlled study (*n* = 18,812) comparing aspirin with extended-release dipyridamole and clopidogrel (the Prevention Regimen for Effectively Avoiding Second Strokes (PRoFESS) trial)	This study aimed to find a possible improvement in cognitive outcomes after stroke with either aspirin, extended-release dipyridamole (ER-DP), clopidogrel, or telmisartan. The authors found that, across all treatment arms, functional impairments were similar. The authors postulate that neither aspirin and ER-DP nor clopidogrel is neuroprotective and that both of these treatments are indeed neuroprotective but yield no changes in functional impairment after stroke. None of the treatments had an influence on cognitive decline and MMSE scores. No change in cognitive outcomes was also found in the smaller subgroup of patients with recurrent stroke. The authors note that the follow-up period of 2 to 5 years in the PRoFESS study may have been too short to properly show the effects of the treatments on cognitive outcomes, especially when juxtaposed to other trials with longer follow-ups that show a greater total decrease in blood pressure and even the reduction of cognitive decline.

13	Price et al. [24]	Scotland	Aspirin 100 mg (*n* = 850) versus placebo (*n* = 858)	Randomized, double-blind, placebo-controlled study (*n* = 1,708) comparing aspirin and placebo	This study, like many before it, investigated the effects of aspirin on cognitive function in those at risk for cardiovascular complications. The authors found that there were no significant differences between those taking aspirin and those on placebo with regard to cognitive function. They were motivated by the experimental evidence that aspirin reduces some cardiovascular disease and can thusly indirectly reduce cognitive decline and the risk of vascular dementia. However, the authors conclude that there is no cognitive benefit seen with aspirin usage in those without dementia and at moderately increased cardiovascular risk. They postulate that a population that is already presented with cognitive decline or with advanced vascular disease, or both, might benefit from such a treatment.

14	Small et al. [25]	California, USA	Celecoxib (*n* = 36) versus placebo (*n* = 36)	Randomized, double-blind, placebo-controlled study (*n* = 72) comparing celecoxib and placebo	This study supports the authors' hypothesis that a selective COX-2 inhibitor like celecoxib can benefit cognitive performance in people with memory complaints when taken daily for 18 months. This study included more middle-aged participants than other usual studies assessing the efficacy of NSAIDs and the cognitive benefits of celecoxib were modest, mainly improving only information processing and retrieval and not episodic memory performance. They bolster these results with PET imaging of glucose metabolism that show increased PET activity in brain regions associated with semantic information processing and retrieval, such as the prefrontal cortex. They note that a number of previous studies state that COX-2 inhibitors can have a null or sometimes negative effect on cognitive performance, but the authors note that the subjects in this study were on average a decade younger than those in the ADAPT study, for example. The younger mean age also contributes to a reduced concern for cardiovascular complications associated with celecoxib and longer administration periods.

15	Szekely et al. [26]	USA	NSAIDs (*n* = 1,180) versus control (no NSAIDs) (*n* = 2,049)	Observational study (*n* = 3,229) via the prospective Cardiovascular Health Cognition Study	This study found that NSAID use was correlated with a reduced risk of dementia and AD. The authors postulate that the protective effects of NSAID use were associated with the APOE status of the subjects, as subjects with multiple APOE epsilon 4 alleles had he least incidence of dementia. Additionally, this study found that the association with AD was seen with neither acetaminophen nor aspirin but the null finding could be due to either a dosage-dependent effect or a misclassification of usage duration (no information on the subject's NSAID usage prior to the study).

16	Waldstein et al. [27]	USA	NSAIDs and aspirin	Observational study (*n* = 2,300) via the prospective Baltimore Longitudinal Study of Aging	This study aimed to investigate the relationship between NSAID and aspirin use and performance on multiple tests of cognitive function in those without dementia. The authors found that NSAID use was related to less decline on tests that assess concentration, memory, and executive function. Aspirin was found to be related to less decline on tests that assessed memory, concentration, and visual memory. Though the actual rate of change of cognitive performance is small, some cross-sectional findings suggest that these medications are neuroprotective, but it is difficult to determine the biological mechanism responsible.

17	Kern et al. [28]	Gothenburg, Sweden	Aspirin (*n* = 129) versus control (no aspirin use) (*n* = 552)	Prospective, population-based cohort study (*n* = 681) of women in Sweden (from the Prospective Population Study of Women and the H70 Birth Cohort Study)	This study aimed to investigate the effect of low-dose aspirin on cognitive function in elderly women with differing cardiovascular risk profiles. This study found that, in women with high risk for cardiovascular disease, low-dose aspirin treatment was linked to less cognitive decline. The authors also note that the use of aspirin did not affect the incidence of dementia, but they owed this null finding to the short follow-up period. The authors also note that the observed increase in cognitive function associated with aspirin use is most likely only seen in those with preclinical dementia, and those with later-stage dementia might not yield as much of a neuroprotective effect.

18	Taguchi et al. [29]	Japan	Cilostazol (*n* = 70) versus control (no cilostazol) (*n* = 22)	Retrospective observational study (*n* = 92) via medical records at Sumotoitsuki Hospital	This study found that cilostazol may preserve cognitive function in those with mild cognitive impairment. In particular, improvements to orientation in time and delayed recall were found, which are critical areas that are first to decline in dementia. The authors postulate that the protective effects of cilostazol come from its dual effects on both AD etiopathogenesis (inhibition of amyloid-beta development) and cerebral perfusion.

19	Ihara et al. [30]	Japan	Donepezil (alone) (*n* = 87) versus combination therapy of donepezil and cilostazol (*n* = 69)	Retrospective observational study (*n* = 156) of subjects in Japan with a history of donepezil use with or without cilostazol use	This study found that cilostazol can preserve cognitive function in patients with mild dementia who are concomitantly taking donepezil for approximately two years. The study's authors hypothesize that the benefit of cilostazol lies in its dual ability to reduce both ischemia and amyloid-beta related neurodegeneration. They also note that the synergistic effects of donepezil and cilostazol can be owed to each drug having different vascular targets: donepezil being endothelium-dependent vasodilation and cilostazol being endothelium-independent vasodilation. One limitation of this study is that it is not clear why one patient would receive cilostazol as an add-on but others would not. Regardless, the study's findings support that cilostazol can delay cognitive decline in patients in early-stage AD or with mild dementia but not in established moderate-to-severe dementia.

20	Zhang et al. [31]	China	Antiplatelet agents (aspirin, clopidogrel, ozagrel) (*n* = 435) versus control (no antiplatelets) (*n* = 83)	Retrospective observational study (*n* = 518) of subjects in China with a diagnosis of acute ischemic stroke	This study found that antiplatelet agents such as aspirin, clopidogrel, and ozagrel reduce the risk of recurrent myocardial and cerebral infarctions and, in general, improve the prognosis of stroke survivors. The authors also found that, in acute ischemic stroke, taking antiplatelet agents shows a favorable functional and cognitive outcome. This effect is nonsignificant, however, when correcting for the use of such agents in the convalescent period of stroke (3 months after stroke). The authors note that, in comparison to other studies examining similar treatments, the population in this study had relatively minor neurologic deficits.

21	Pearce et al. [32]	81 clinical centers worldwide	Aspirin and clopidogrel (*n* = 1,468) versus aspirin and placebo (no clopidogrel) (*n* = 1,448)	Randomized, double-blind, placebo-controlled study (*n* = 2,916) (Secondary Prevention of Small Subcortical Strokes (SPS3) trial)	This study investigated the effects of dual antiplatelet therapy with aspirin and clopidogrel in patients with cerebral small vessel disease, a history of lacunar strokes, and their effects on cognition. The study's findings suggest that, for these patients (in the absence of a recurrent stroke), adequate management of blood pressure and a dual antiplatelet regimen leads to minimal cognitive decline in a short term (3 years). However, no clinically significant difference was found between the dual antiplatelet plus clopidogrel group and the antiplatelet plus placebo group.

22	Wichmann et al. [33]	Wisconsin, USA	Aspirin (*n* = 1,583) and nonaspirin NSAIDS (*n* = 1,111) versus control (no aspirin nor NSAIDs) (*n* = 1,965)	Observational study via the prospective Epidemiology of Hearing Loss Study of patients with mild cognitive impairment and sensory loss; participants without cognitive impairment at baseline	This study's findings do not support NSAID's protective effect against cognitive impairment. Aspirin use was related to increased risk of cognitive impairment, possibly due to indication bias. Those who used aspirin at baseline but not 5 years before had a higher risk of cognitive impairment over the next decade. The current study found an elevated risk of cognitive impairment among aspirin users but only among current users at baseline. This study assessed NSAID use and incident cognitive impairment over a 20-year period and found no indication of a protective effect. Debate continues over nonaspirin NSAID use and dementia risk because most observational research yields contradictory results.

23	Chang et al. [34]	Taiwan	Aspirin (*n* = 2,876) versus control (no aspirin) (*n* = 10,720)	Retrospective cohort study (*n* = 28,321) of patients with type 2 diabetes mellitus without a prior diagnosis of dementia	A daily dose of 40 mg aspirin may reduce the incidence of Alzheimer's Disease in people with type 2 diabetes but not in people with non-Alzheimer's dementia. The absence of statistical significance of low-dose aspirin's protective benefits against non-Alzheimer's dementia was likely owing to small sample size. The authors additionally found that increasing the dosage of aspirin greater than 80 mg/day increased the risks of both non-AD and AD in those with T2DM.

24	Oveisgharan and Hachinski [35]	Canada	Unspecified salicylate therapy	Observational study (*n* = 4,878) of patients with cognitive impairment via the longitudinal Canadian Study of Health and Ageing	This study suggests that salicylate use may reduce dementia risk in older people who score poorly on a basic neuropsychological test and are 2 to 3 times more likely to acquire dementia than people with normal cognition. The authors additionally found that there was no link between incident dementia amongst normal cognition patients and the use of salicylates; however, for subjects with normal copying pentagons-writing scores in the studied neuropsychological tests, salicylates enhanced dementia incidence. It is probable that some of these people had preclinical AD diseases such amyloid angiopathy, and salicylates have been linked to brain microbleeds, which are linked to dementia.

25	Tai et al. [36]	Taiwan	Cilostazol (*n* = 2,287) versus control (*n* = 6,861)	Prospective cohort study (*n* = 9,148) of patients who had ever taken cilostazol for at least 3 months via Taiwan's National Health Research Institute	This study found a significant link between a decreased risk of dementia in those without a prior history of dementia and the use of cilostazol. The authors note that this relationship was dose-dependent (high-dose cilostazol significantly decreased the risk of dementia). They additionally find that cilostazol use was notable for reducing the risk of dementia in those with vascular pathologies like ischemic heart disease and cerebral vascular disease and that this neuroprotective effect was notably seen in men older than 65 years.

26	Staszewski et al. [37]	Poland	Aspirin	Observational study of patients with first-ever LS (*n* = 49), diagnosed VP (*n* = 16), and VD (*n* = 39) due to CSVD	This study's main conclusion is that AR is frequent in CSVD patients and is linked to radiological progression and lacunar stroke risk. The prevalence of AR was similar in patients with acute (lacunar stroke) and chronic (VD and VP) CSVD. They found no difference in ASA treatment duration between AR and RTA patients. A similar frequency of AR was found in individuals who received ASA prior to study admission and those who started ASA after trial entry. Those with VD or VP had a higher risk of vascular events attributable to age (mean 72 years), hypertension and dyslipidemia, and diabetes than those without VD or VP. The authors also found that TG levels were linked to AR regardless of statin use (patients with AR had slightly higher TG).

27	Ding et al. [38]	Stockholm, Sweden	Unspecified anticoagulation and antiplatelet therapy	Population-based cohort study with patients at risk for either VD or AD due to CVD (total *n* = 2,685) and those specifically with AF (*n* = 243)	This population-based investigation found that anticoagulant but not antiplatelet therapy lowered the risk of dementia in AF patients. The study's authors also note that AF in the elderly is associated with an increased risk of cognitive decline and dementia. Because of this somewhat novel association between these two pathologies, this study motivates the need for trials investigating the effects of using antithrombotics and anticoagulants for the management of dementia. The authors hypothesize that the link between AF and dementia is due to reduced cardiac output, increased blood stasis, blood hypercoagulability, and the increased risk of cerebrovascular lesions, factors that are often targeted by the novel direct oral anticoagulants.

28	Yang et al. [39]	Taiwan	Aspirin (*n* = 1,525) and control (*n* = 1,525)	Population-based study (*n* = 6,028) of patients with cognitive impairment and LOD via the National Health Insurance of Taiwan survey	This study shows that aspirin consumption reduces the risk of dementia and also provides evidence that LOD is a risk factor for dementia. The authors note that, in this study, like many studies before it investigating the effects of aspirin in the elderly with dementia, many of the subjects copresented with cardiovascular disease, especially those with LOD, and this association possibly explains the protective effects of aspirin. Their findings should be taken with caution in light of the ASPREE trial's poor findings on low-dose preventative aspirin use in healthy older people; nevertheless, the authors maintain that aspirin might still be beneficial for thrombosis prevention in the elderly with dementia, cardiovascular risk, and LOD, especially in those who do not present with the increased hemorrhagic risk of aspirin.

29	Viticchi et al. [40]	Italy	Unspecified antiplatelet and anticoagulation therapy	Retrospective cohort analysis of hospitalized patients with preexistent AF (total *n* = 1,705) and those with VD (*n* = 193) via the Atrial Fibrillation in Critically Ill (AFICILL) study	This study aimed to evaluate the effect of compliance and adherence to antiplatelet and anticoagulant therapy in patients with dementia and atrial fibrillation. Primarily, the authors found that antiplatelets and anticoagulants are often underadministered or not given to patients where these medications are indicated. They also found that patients with other forms of dementia were also undertreated, albeit less so than VD patients. Moreover, VD patients had a higher incidence of acute cerebrovascular disorders during hospitalization, with some having a new stroke/TIA, compared to patients with other forms of dementia. VD patients had higher thromboembolic risk scores than non-VD patients. Despite this, VD patients had a lower rate of major bleeding and a greater rate of stroke. They also showed no significant difference in treatment failure compared to nondemented patients. The authors conclude that the risk of bleeding in the elderly can lead to undertreatment, especially those affected by dementia, and this leads to a source of understudied treatment effects on this patient population.

30	Matsumoto et al. [41]	Japan	Aspirin (*n* = 1,259) versus control (no aspirin) (*n* = 1,277)	Follow-up cohort study (*n* = 2,536) of patients with type 2 diabetes mellitus (Japanese Primary Prevention of Atherosclerosis with Aspirin for Diabetes (JPAD))	This study demonstrates that low-dose aspirin may help prevent dementia in T2D women but not necessarily in men. This study is the first to use real-world data to assess the long-term relationship between low-dose aspirin and dementia risk in T2D patients. The authors postulate that aspirin's dementia-preventive effects may be strengthened in people with cardiovascular risk. They note this as a possible source of bias especially since dementia and cardiovascular disease share common factors in their etiopathogenesis. This study also features a longer follow-up (11.4 years) than most other studies and could be a reason for the positive finding not seen in shorter aspirin related trials.

31	Ryan et al. [42]	USA and Australia	Aspirin (*n* = 9,525) versus placebo (*n* = 9,589)	Randomized, double-blind, placebo-controlled study (*n* = 19,114) of patients with no history of CVD but diagnosed with dementia (Aspirin in Reducing Events in the Elderly (ASPREE))	In ASPREE, they found that daily low-dose aspirin did not improve disability-free survival but increased the chance of severe hemorrhage. Low-dose aspirin had no effect on dementia incidence. On average, the treatment groups did not benefit from aspirin when it came to clinically probable or plausible AD, MCI, or cognitive impairment. Aspirin had no effect on global or specific cognitive domains. Treatment results did not differ between subgroups characterized by age, sex, ethnicity, health, or prior NSAID usage. Because of the rigor of this large double-blinded placebo-controlled trial, this study provides good evidence that starting low-dose aspirin for those older than 70 years does not reduce the risk of cognitive decline, dementia, or AD in older adults.

32	Khezrian et al. [43]	Scotland	Aspirin (*n* = 68) versus control (no aspirin) (*n* = 250)	Longitudinal observational study (*n* = 318) via the Aberdeen 1936 Birth Cohort study	This study aimed to describe the relationship between white-matter hyperintensity total lesion volumes, cardiovascular risk, cognitive decline, and the administration of aspirin. This study found that aspirin is expectedly beneficial for the prevention of recurrent cardiovascular incidents such as stroke or MI but can be detrimental for information processing speed; they find that this negative effect of aspirin is exacerbated by white matter hyperintensity burden. The authors conclude that, in future analyses of the relationship between cardiovascular risk and cognitive decline, assessments of white matter hyperintensity volumes should be included, which was shown to be a significant predictor of cognitive decline and, in particular, information processing speed.

33	Huang et al. [44]	Southern Taiwan	Cilostazol (*n* = 45) versus aspirin or clopidogrel (*n* = 45)	Prospective open-label study (*n* = 90) of subjects with a diagnosis of cognitive impairment after ischemic stroke	This study describes poststroke cognitive alterations. The cilostazol and control groups had similar changes in cognitive and global status and the study concludes that cilostazol did not make a significant difference in global cognitive functioning relative to other antiplatelet drugs. After 6 months, over 60% of patients improved their cognitive function and over 70% improved their global status in both treatment arms. Cilostazol may be regarded as “noninferior” for poststroke cognitive change due to the bias of higher recruiting into the cilostazol group for the treatment of PAOD, which many of the subjects copresented with. Cilostazol is recommended by Taiwan's national health insurance standards for treating PAOD symptoms of intermittent claudication and thus many subjects were recruited into the cilostazol treatment arm.

34	Lee et al. [45]	Korea	Warfarin (*n* = 25,948) versus DOACs (*n* = 46,898) (rivaroxaban, dabigatran, apixaban, edoxaban)	Observational study (*n* = 72,486) via the Korean nationwide claims database	Compared to warfarin, DOACs had equivalent dementia, vascular dementia, and Alzheimer's dementia risks. For individuals aged 65 to 74 yesars and those who had previously had a stroke, DOACs were associated with a decreased incidence of dementia than warfarin. When compared to warfarin, edoxaban and rivaroxaban had a decreased risk of dementia.

35	Kim et al. [46]	Korea	Cilostazol (*n* = 127) versus aspirin (*n* = 129)	Randomized, double-blind, controlled study (*n* = 256) comparing cilostazol and aspirin	This study aimed to investigate any differences seen in white matter changes in cerebral small vessel disease, such as lacunar infarctions, when receiving cilostazol or aspirin. Both groups, over the two-year period of the trial, had increased WMH volume to total WM volume, but the treatment arms showed no significant differences. The cilostazol group had reduced ischemic vascular events relative to the aspirin group, but this was a secondary outcome.

## Data Availability

No data were used in this article as this is a systematic review.

## Abbreviations

AD: Alzheimer's Disease
AF: Atrial fibrillation
AR: Aspirin resistance
ASA: Aspirin
ASPREE: Aspirin in reducing events in the elderly clinical trial
CSVD: Cerebral small vessel disease
COX-1 or COX-2: Cyclooxygenase enzyme 1 or 2
CVD: Cardiovascular disease
DOAC: Direct oral anticoagulant
HLA: Human leukocyte antigen
LOD: Late-onset depression
LS: Lacunar stroke
MCI: Mild cognitive impairment
MI: Myocardial infarction
MMSE: Mini-mental status exam
NSAID: Nonsteroidal anti-inflammatory drug
PAOD: Peripheral arterial occlusive disease
PET: Positron emission tomography
RTA: Responders to aspirin
T2DM or T2D: Type 2 diabetes mellitus
TG: Triglyceride or triglyceride levels
TIA: Transient ischemic attack
TICS: Telephone interview for cognitive status
VD: Vascular dementia
VP: Vascular Parkinsonism
WMH: White matter hyperintensity
WM: White matter.
